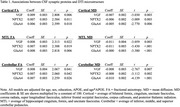# Cerebrospinal fluid synaptic markers are associated with global white matter microstructure in cognitively unimpaired participants

**DOI:** 10.1002/alz70862_109914

**Published:** 2025-12-23

**Authors:** Elizabeth R Paitel, Corinne Pettigrew, Abhay Moghekar, Daniel D. Callow, Claire L Anderson, Chan‐Hyun Na, Paul F Worley, Marilyn S. S. Albert, Anja Soldan

**Affiliations:** ^1^ Johns Hopkins University School of Medicine, Baltimore, MD USA; ^2^ Department of Neurology, Johns Hopkins University School of Medicine, Baltimore, MD USA

## Abstract

**Background:**

Higher levels of the synaptic proteins neuropentraxin 2 (NPTX2) and neurosecretory protein VGF in cerebrospinal fluid (CSF) are associated with reduced cognitive decline among older adults, but the mechanisms underlying their beneficial effects on cognition are poorly understood. The present study examined the cross‐sectional relationship between levels of three synaptic proteins (NPTX2, VGF, and a synaptic glutamate receptor subunit (GluA4)) with MRI measures of white matter microstructure, and whether that relationship is influenced by CSF AD biomarker levels.

**Method:**

CSF and MRI data were collected from 126 cognitively unimpaired middle‐age and older adult participants (*M*_age_=68.6, *SD*_age_=8.7, range=34‐89) from the BIOCARD study. Synaptic proteins were measured in CSF from two to three highly correlated peptides using quantitative mass spectrometry and averaged into composite scores. CSF AD biomarker levels were measured using automated electrochemiluminescence assays and summarized as the ratio of *p*‐tau_181_ to Aβ_42_/Aβ_40_. White matter microstructure (fractional anisotropy (FA), mean diffusion (MD)) from diffusion tensor imaging (DTI) was assessed in three composite tracts: global cerebral cortex, medial temporal lobe, and cerebellar peduncles. Linear regressions assessed associations of microstructure measures with the synaptic proteins, interactions with AD biomarker levels, and associations with cognition (episodic memory, executive function, and visuospatial composites). Models covaried age, sex, education, APOE‐ε4, and age*APOE‐ε4.

**Result:**

Higher values of all three synaptic proteins were associated with better DTI microstructure (i.e., higher FA, lower MD) across all regions (Table 1). The patterns remained unchanged with CSF AD biomarkers included as additional model predictors, and there were no interactions with AD biomarker levels. Additionally, better DTI microstructure in cortical and cerebellar tracts was associated with better executive function and visuospatial performance.

**Conclusion:**

Higher CSF levels of synaptic markers NPTX2, GluA4, and VGF were associated with better white matter integrity, including global cortical, medial temporal, and cerebellar tracts, which in turn was associated with better cognitive performance. These results support the view that synaptic proteins may contribute to cognitive resilience among older adults, possibly via better white matter microstructure. Longitudinal studies are needed to assess the impact of these proteins on white matter microstructure and their relationship with cognition.